# Arterial communication around the pancreatic tail enabled division of the gastroduodenal artery during pancreaticoduodenectomy in patient with complete celiac artery occlusion: a case report

**DOI:** 10.1186/s40792-020-0787-2

**Published:** 2020-01-28

**Authors:** Ryo Oikawa, Kyoji Ito, Nobuyuki Takemura, Fuminori Mihara, Norihiro Kokudo

**Affiliations:** Hepato-Biliary-Pancreatic Surgery Division, Department of Surgery, National Center for Global Health and Medicine, 1-21-1 Toyama, Shinjuku-ku, Tokyo, 162-8655 Japan

**Keywords:** Celiac artery stenosis, Pancreaticoduodenectomy, Three-dimensional imaging, Gastroduodenal artery clamping test, Peri-pancreatic arterial communication

## Abstract

**Background:**

Stenosis or obstruction of the celiac artery (CA) is known as celiac artery stenosis (CAS) and is usually accompanied by the formation of arterial anastomosis between the superior mesenteric artery (SMA) system and the CA system. Arterial bypass is mainly achieved through the gastroduodenal artery (GDA); therefore, the division of the GDA during pancreaticoduodenectomy (PD) could pose a problem in patients with CAS.

**Case presentation:**

We reported a case of PD presenting complete occlusion of the CA, in which perfusion to organs in the CA system was maintained via peri-pancreatic arterial communication. There were complicated arterial anastomoses around the pancreas, which were clearly visualized on a three-dimensional reconstruction of the arterial system using multi-detector computed tomography. Among these complicated anastomoses, one well-developed anastomosis between the SMA and the splenic artery through the dorsal pancreatic artery (DPA) was identified. The DPA was considered to work as a potential collateral pathway from the SMA to organs in the CA system after division of the GDA. During surgery, Doppler ultrasonography detected hepatopetal arterial flow even after the GDA clamping; therefore, we performed typical PD with division of the GDA. The postoperative course of the patient was uneventful, and there was no sign of ischemic complications in the CA system organs including the liver, stomach or spleen.

**Conclusions:**

Three-dimensional reconstruction of the arterial system using multi-detector computed tomography and the intraoperative GDA clamping test were useful to determine whether it was possible to divide the GDA in PD, in the case of CAS.

## Background

The celiac artery (CA) is the first major branch of the abdominal aorta supplying blood to the liver, stomach, spleen, duodenum, and pancreas. Stenosis or obstruction of the CA, known as celiac artery stenosis (CAS), can result from various factors, including median arcuate ligament syndrome (MALS), atherosclerosis, and invasion of malignancies or pancreatitis [1]. However, CAS is often asymptomatic due to the formation of collateral blood flow from surrounding arteries [2]. In many cases, the gastroduodenal artery (GDA) is a main collateral pathway from the superior mesenteric artery (SMA) to the CA system through common hepatic artery (CHA) [2–5].

Pancreaticoduodenectomy (PD) is a surgical procedure to resect pancreatic head tumor, including pancreatic adenocarcinoma, distal bile duct cancer, and ampullary carcinoma [6]. The procedure of PD includes the division of the GDA, and therefore, CAS becomes a big issue in PD, because the division of the GDA could threaten the hepatobiliary, gastric, and splenic arterial circulation normally supplied directly by the CA [7]. To prevent ischemia of these organs after PD, interventional radiology, arterial reconstruction, and median arcuate ligament division surgery are reportedly considered [8]. Alternatively, several studies indicated low incidence of ischemic complications after typical PD without any preoperative intervention or reconstruction of the GDA in patients with CAS [7, 9]. In this report, we described a case of PD presenting complete occlusion of the CA, in which a preoperative three-dimensional (3D) reconstruction of the arterial system using multi-detector computed tomography (MDCT) and the intraoperative GDA clamping test were useful to determine whether it was possible to divide the GDA.

## Case presentation

**Fig. 1:**
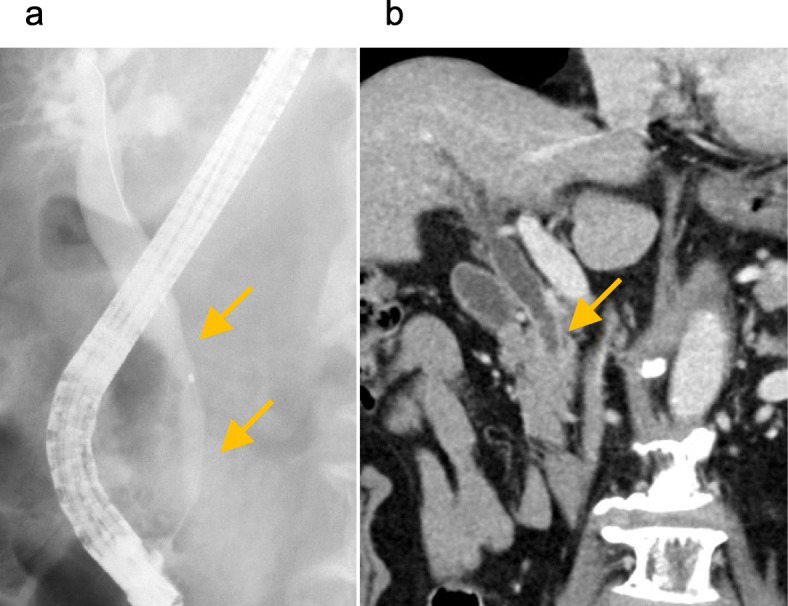
Images of endoscopic retrograde cholangiography and MDCT of the distal bile duct. **a** Endoscopic retrograde cholangiography. Narrowing and wall-irregularity of the distal bile duct were found (arrow). **b** Abdominal MDCT. Wall thickness with contrast enhancement of the distal bile duct was identified (arrow). There was no invasion to the surrounding organs or vessels. MDCT, multi-detector computed tomography

**Fig. 2:**
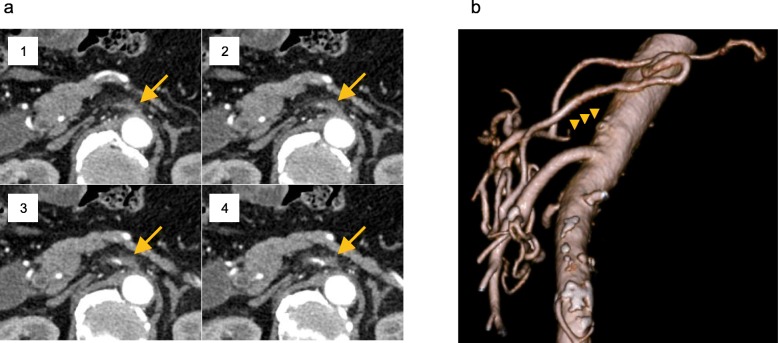
Serial axial view and three-dimensional reconstruction of the CA using the preoperative MDCT. **a** Serial axial view of the preoperative MDCT. Complete occlusion of the CA was found (arrow). Images are shown from the cranial to the caudal section in the order from 1 to 4. **b** Three-dimensional reconstruction of the preoperative MDCT. The CA was completely disrupted (arrow heads). CA, celiac artery; MDCT, multi-detector computed tomography

**Fig. 3:**
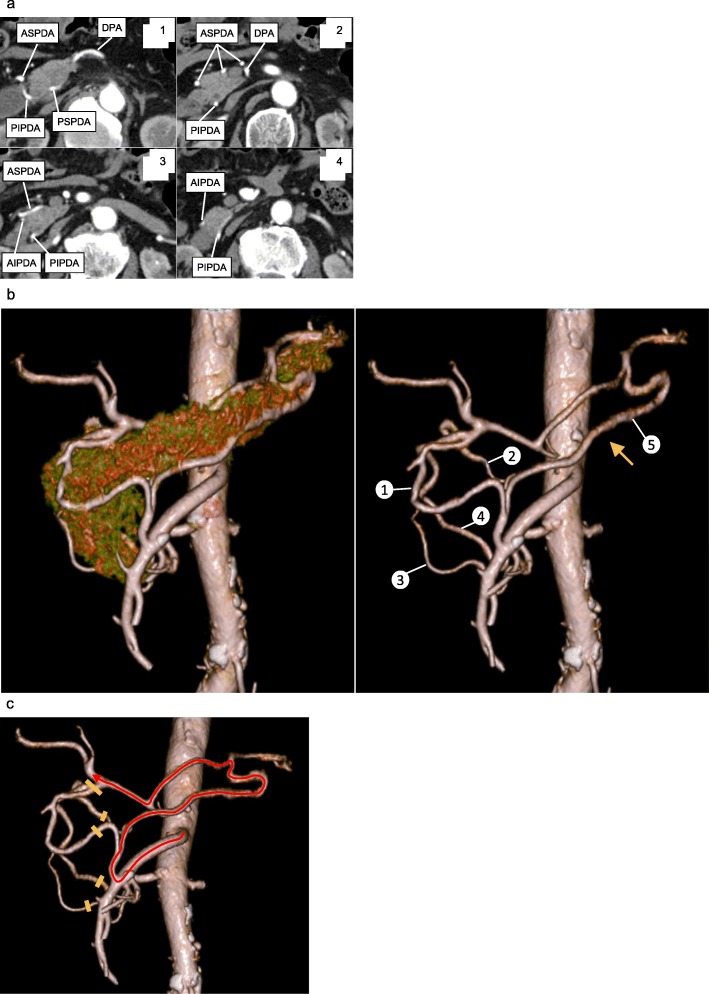
Serial axial view and three-dimensional reconstruction of peri-pancreatic arterial anastomoses on the preoperative MDCT. **a** Serial axial view of the preoperative MDCT. Complicated arterial anastomoses from the SMA system to the CA system were detected. Images were shown from cranial to caudal section in the order from 1 to 4. **b** Three-dimensional reconstruction of the pancreas and artery (left) and the artery alone (right) using the preoperative MDCT. Well-developed anastomoses between the SMA and CHA through GDA, including the ASPDA (1), PSPDA (2), AIPDA (3), and PIPDA (4), were clearly detected. In addition, one well-developed anastomosis between SMA and SPA through DPA was also identified (5, arrow). **c** The remaining anastomosis and arterial flow from the SMA to the CHA through the DPA after division of the GDA during PD. AIPDA, anterior inferior pancreaticoduodenal artery; ASPDA, anterior superior pancreaticoduodenal artery; CA, celiac artery; DPA, dorsal pancreatic artery; GDA, gastroduodenal artery; MDCT, multi-detector computed tomography; PD, pancreaticoduodenectomy; PIPDA, posterior inferior pancreaticoduodenal artery; PSPDA, posterior superior pancreaticoduodenal artery; SMA, superior mesenteric artery; SPA, splenic artery

An 80-year-old man with epigastric pain visited our hospital in January 2019. A magnetic resonance cholangiopancreatography revealed several stones in the common bile duct (CBD), and he was diagnosed with choledocholithiasis. Endoscopic lithotripsy was performed, and cholangiography showed narrowing and wall-irregularity of the lower CBD (Fig. 1a). Brushing cytology detected class V malignant cells, and laboratory findings showed elevation of carcinoembryonic antigen, 8.2 ng/mL (0.0–4.9 ng/mL). Abdominal MDCT showed wall thickness and contrast enhancement of the lower CBD. Invasion to the surrounding organs or vessels was not detected, and there was no significant lymph node swelling or remote metastasis (Fig. 1b). He was diagnosed with distal bile duct cancer of T1bN0M0 Stage IA according to the Japanese Society of Hepato-Biliary-Pancreatic Surgery classification [10], and PD was scheduled. On the preoperative MDCT images, complete occlusion of the CA was incidentally detected (Fig. 2a, b). There was considerable calcification around the occlusion of the CA; therefore, the cause of the occlusion was suspected to be arteriosclerosis. MDCT images identified complicated peri-pancreatic arterial anastomoses from the SMA to the CA system (Fig. 3a). A 3D image of the arterial system was reconstructed from MDCT images, and the arterial anastomoses were clearly visualized and easily detected (Fig. 3b). Although most of the anastomoses were developed through the GDA, we identified one well-developed anastomosis between the SMA and the splenic artery (SPA) through the dorsal pancreatic artery (DPA) (Fig. 3b, arrow). Preoperative abdominal angiography was also considered to evaluate collateral arteries and perform preoperative TAE of the GDA because of the complete obstruction of the CA. However, it was assumed that the arterial blood flow to the spleen, stomach, and liver could be supplied via the anastomosis from the SMA to the SPA through the DPA, even if we divided the GDA (Fig. 3c). In order to confirm this hypothesis, we planned to perform a GDA clamping test during the surgery and check the blood flow of the hepatic artery with intraoperative ultrasonography to determine whether it was possible to divide the GDA. In the case of deficient blood flow of the hepatic artery after the GDA clamping test, we scheduled reconstruction of the GDA using the middle colic artery by plastic surgeons, or the preservation of arterial anastomosis between the SMA and the GDA around the pancreatic head when possible.

**Fig. 4:**
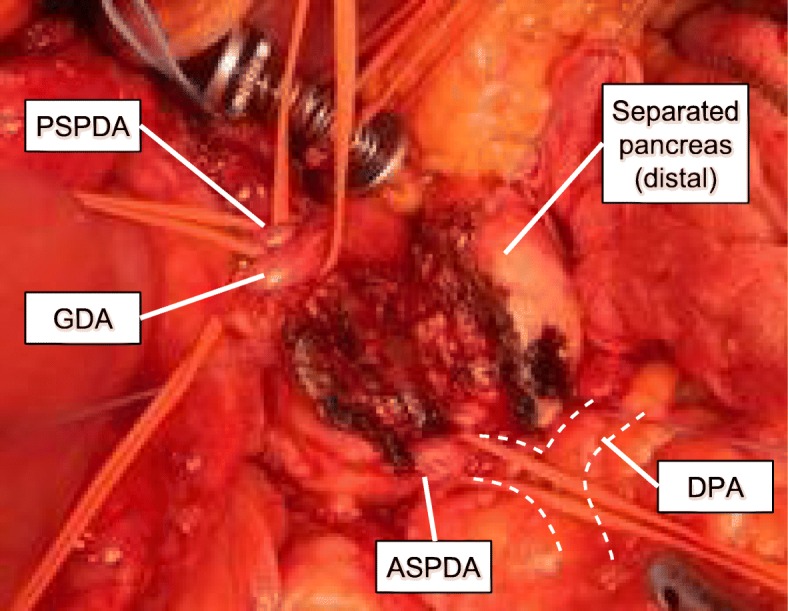
Intraoperative photograph after taping the ASPDA, GDA, and PSPDA. Enlarged arterial communications around the pancreas were identified. The position of the DPA was confirmed by palpation and intraoperative ultrasonography. ASPDA, anterior superior pancreaticoduodenal artery; DPA, dorsal pancreatic artery; GDA, gastroduodenal artery; PSPDA, posterior superior pancreaticoduodenal artery

**Fig. 5:**
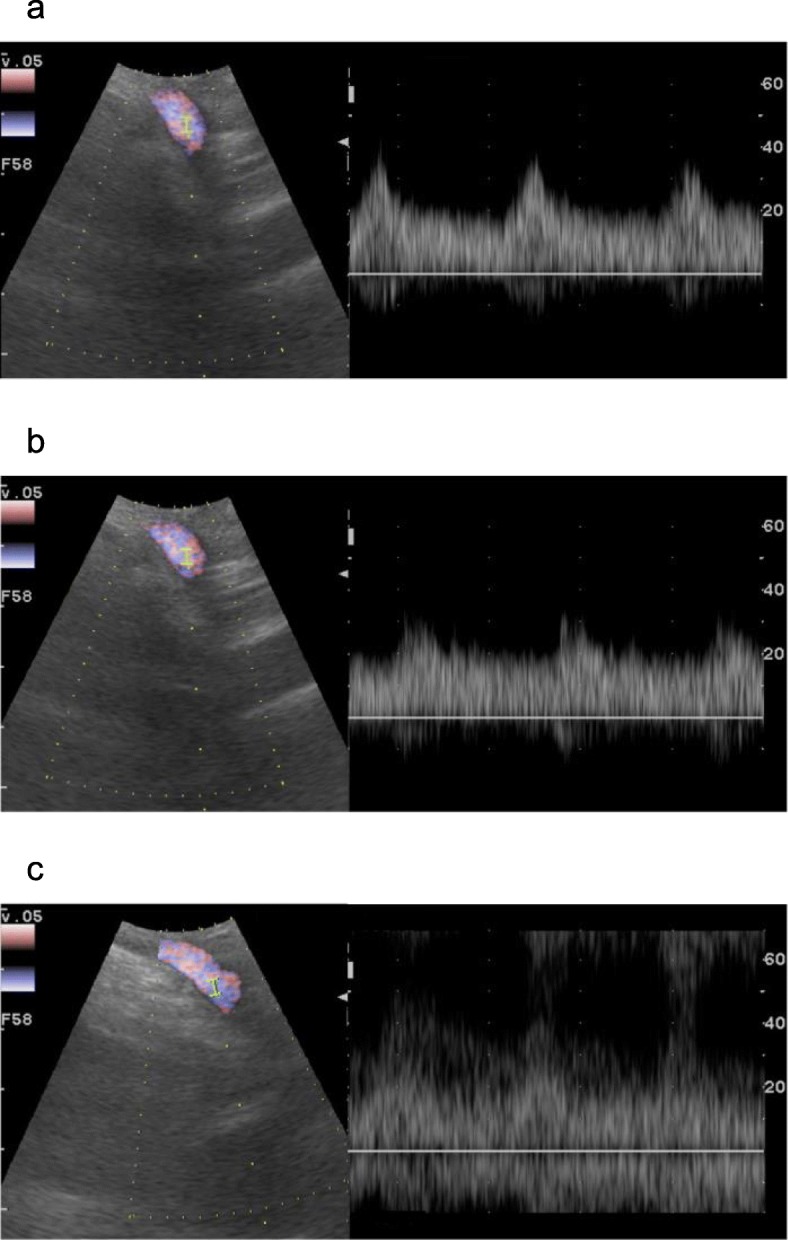
Assessment of the hepatic arterial blood flow with intraoperative doppler ultrasonography. **a** Hepatic arterial blood flow before the GDA clamping test. The acceleration time was 143 ms and resistive index was 0.531. **b** Hepatic arterial blood flow after the GDA clamping test. Acceleration time decreased to 60 ms and resistive index decreased to 0.428, suggesting no tardus-parvus pattern with the GDA clamping. **c** Hepatic arterial blood flow at the end of the operation. Acceleration time was 144 ms and resistive index was 0.537, suggesting no tardus-parvus pattern. GDA, gastroduodenal artery

PD was performed in April 2019. After taping of the GDA (Fig. 4), CHA, and proper hepatic artery (PHA), we clamped the GDA, and checked the arterial flow in the PHA using Doppler ultrasonography (Fig. 5). Although the acceleration time decreased from 143 to 60 ms and the resistive index decreased from 0.531 to 0.428 after the clamping test [11], a tardus-parvus pattern was not detected with the clamping test [12], suggesting the arterial anastomosis from the SMA to the PHA through the DPA and the SPA worked well. We divided the GDA and performed a typical PD without any additional complicated procedures [6], including arterial reconstruction and the preservation of arterial anastomoses between the SMA and the CHA around the pancreatic head. At the end of the operation, we checked the blood flow in the PHA once again. The acceleration time was 144 ms and the resistive index was 0.537, suggesting absence of a tardus-parvus pattern. There were well-developed arterial anastomoses around the pancreatic head. Especially, arteries in the mesopancreas (pancreatic nerve plexus) were quite developed and thus, resection of the mesopancreas was excessively hemorrhagic. The operative time was 6 h and 40 min, and the estimated blood loss was 1377 ml. Pathological diagnosis was distal bile duct tubular adenocarcinoma of T3(SS), N1, Stage IIB [10].

**Fig. 6:**
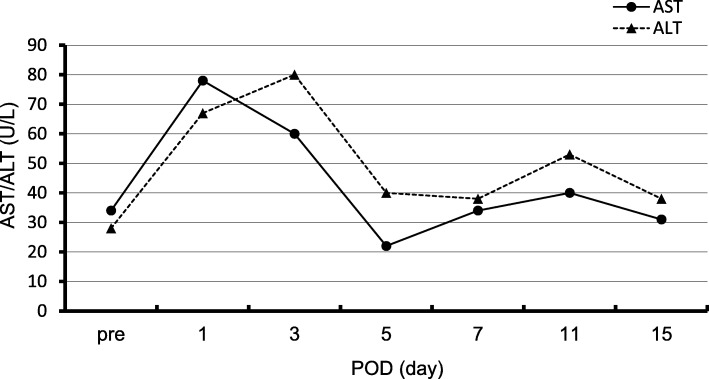
Transition of hepatic enzymes in the pre and postoperative blood tests. There was only slight elevation of hepatic enzymes after the surgery. ALT, alanine aminotransferase; AST, aspartate aminotransferase

**Fig. 7:**
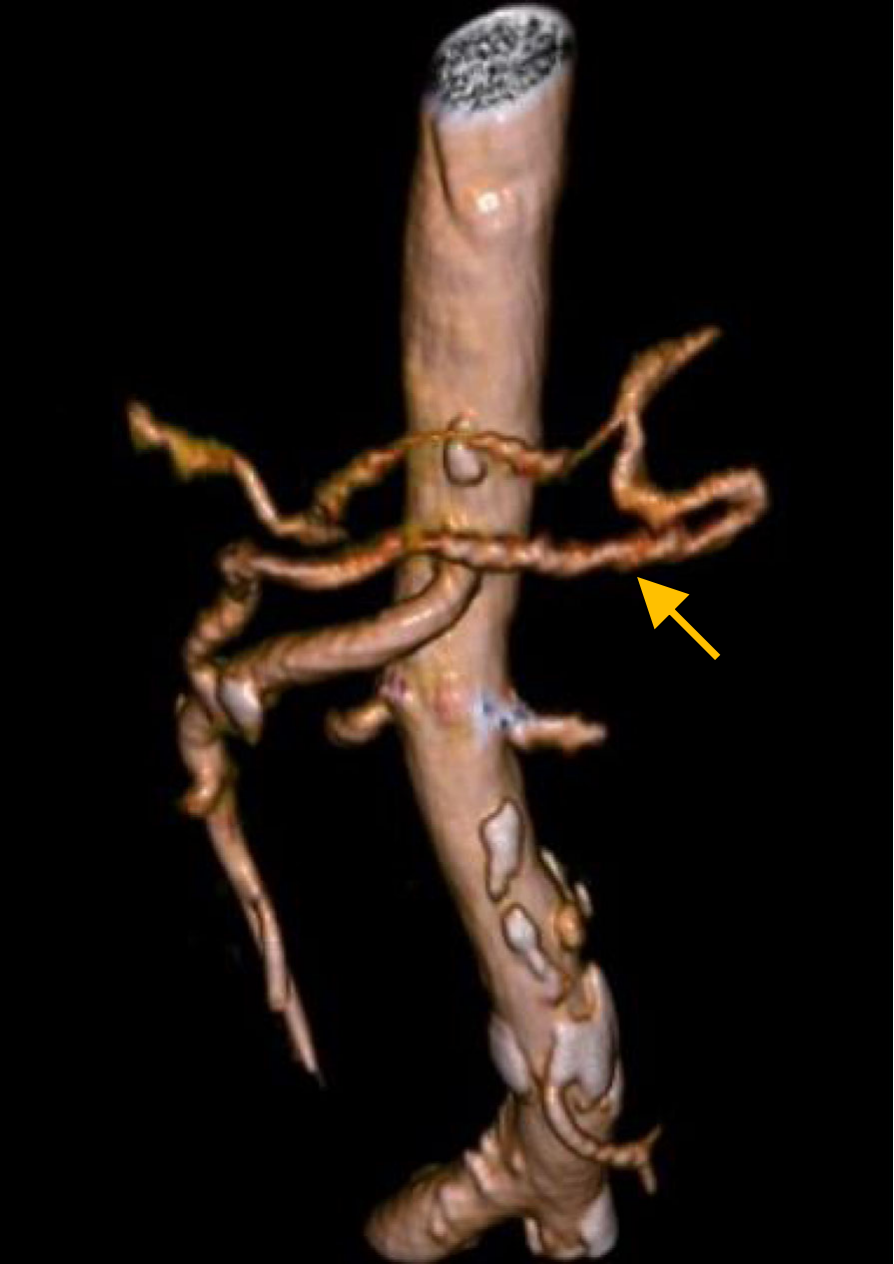
Three-dimensional reconstruction of the peri-pancreatic arterial communication on the postoperative MDCT. The anastomosis between the SMA and SPA through the DPA was clearly detected (arrow). DPA, dorsal pancreatic artery; MDCT, multi-detector computed tomography; SMA, superior mesenteric artery; SPA, splenic artery

Postoperative laboratory data showed slight elevation of hepatic enzyme immediately after the surgery (Fig. 6), but we did not observe jaundice or increase of ascites after the surgery, suggesting no signs of ischemia of the liver. There was also no delayed gastric emptying, which is sometimes caused by ischemia of the stomach after PD [8, 13, 14]. Pancreatic fistula of Grade B [15] developed, and it was resolved without any invasive intervention. The patient was discharged from the hospital 41 days after surgery. The postoperative MDCT 3 months after surgery detected arterial perfusion to the CHA from the SMA via the DPA and SPA (Fig. 7).

## Discussion

CAS is a relatively common finding, identified in 12.5–24% of patients undergoing abdominal angiography [1, 16]. The typical symptoms of CAS are weight loss, postprandial abdominal pain, or nausea and vomiting [17, 18], although most patients with CAS are asymptomatic unless the perfusion decreases by 60 to 75% [1, 4]. The most common causes of CAS are MALS in Eastern countries and atherosclerosis in Western countries [7, 19]. In addition, congenital causes, acute and chronic aortic dissection, pancreatitis, invasion of malignancies, or iatrogenicity are known as causes of CAS [1]. In the present case of the complete CA occlusion, the preoperative MDCT did not detect a lower location of the median arcuate ligament [3] or direct invasion of the CA by the tumor, and the patient did not have a past medical history of aortic dissection and pancreatitis. Instead, there was severe arteriosclerosis throughout the systemic arteries, including the CA; therefore, the cause of complete occlusion of the CA in the patient was considered to be atherosclerosis.

Severe stenosis or obstruction of the CA often induces the formation of collateral arterial pathways from the SMA system to the CA system to compensate the decrease of splanchnic arterial flow of the upper abdomen. The main collateral pathway is reported to be the GDA or DPA in most cases, whereas various arteries, such as the DPA, jejunal artery, omental artery, left gastric artery, arc of Buhlar, and Riolan or Drummond artery can be a collateral pathway [1, 5]. Song et al. described a total of 181 anastomoses in 94 CAS patients, including 89 pancreaticoduodenal arcades and 71 dorsal pancreatic arcades [2]. Heo et al. described 22 collateral circulations in 37 MALS cases, including 14 pancreaticoduodenal arcades and 2 dorsal pancreatic arcades. The other 6 arcades were found in the arc of Buhler, splenic artery, jejunal artery, omental artery, hepatic artery, and left gastric artery [4]. Therefore, the evaluation of the collateral pathways in patients with CAS is critical, especially before upper abdominal surgeries, to avoid organ ischemia. In the present case, the complete occlusion of the CA and the formation of complicated collateral arterial pathways were found in the two-dimensional MDCT images. In order to identify the accurate collateral pathways, a 3D image was reconstructed from MDCT and we clearly detected enlarged arteries around the pancreatic head, including the anterior superior pancreaticoduodenal artery (ASPDA), the posterior superior pancreaticoduodenal artery, the anterior inferior pancreaticoduodenal artery, and the posterior inferior pancreaticoduodenal artery, which perfuse from the SMA to the CHA through the GDA. In addition, we identified the well-developed DPA as a substantial anastomosis from the SMA to the SPA, suggesting that the DPA could function as one of the collateral arterial pathways from the SMA system to the CA system.

In the context of CAS, the formation of collateral arterial pathways is surgically relevant for arterial division in PD. Although the GDA is the main collateral pathway from the SMA system to the CA system in most of the cases of CAS, the GDA must be divided in PD. Therefore, various procedures were attempted to avoid organ ischemia after PD in patients with CAS [1]. There are numerous reports regarding interventional radiological procedures that describe stenting of the CA for CAS with atherosclerosis [20]. Various methods of vascular reconstruction during surgery were also proposed [1], including bypass between aorta or SMA and CA system using saphenous vein or prosthetic vascular graft [7, 21–23], bypass between SPA and iliac artery using autogenous vein [22], GDA reconstruction using middle colic artery [24], reimplantation of celiac trunk into the aorta [25], and arterial reimplantation of the proximal end of the divided SPA into the side of SMA [26]. Median arcuate ligament division surgery is also considered as an effective method for CAS caused by MALS [17]. In the present case, we identified the DPA as a collateral pathway from the SMA to the SPA, and the DPA is assumed to perfuse the CA system after division of the GDA. To confirm this hypothesis, we performed a GDA clamping test during the surgery, and favorable arterial waveform in the PHA was detected, suggesting that the arterial blood supply in the CA system was secured by the DPA. Indeed, a typical PD with GDA division was performed for this patient, and we did not find any signs or symptoms of upper abdominal organ ischemia, including the liver and stomach after the surgery. Although we used only Doppler ultrasonography to confirm arterial flow in the liver after GDA clamping, ICG fluorescence would also be useful to evaluate the arterial flow via collateral pathways in this case [27]. The PD procedure performed for this patient with CAS was simple and easy to implement.

## Conclusions

In conclusion, we described a case of PD with complete occlusion of the CA, in which we maintained perfusion via the peri-pancreatic arterial communication. A 3D reconstruction of the arterial system from MDCT and intraoperative GDA clamping test were useful to determine whether it was possible to divide the GDA.

## Data Availability

The datasets analyzed during the current study are not publicly available due to their containing information that could compromise the privacy of research participants but are available from the corresponding author on reasonable request.

## Abbreviations

ASPDA: Anterior superior pancreaticoduodenal artery
CA: Celiac artery
CAS: Celiac artery stenosis
CBD: Common bile duct
CHA: Common hepatic artery
DPA: Dorsal pancreatic artery
GDA: Gastroduodenal artery
MALS: Median arcuate ligament syndrome
MDCT: Multi-detector computed tomography
PD: Pancreaticoduodenectomy
PHA: Proper hepatic artery
SMA: Superior mesenteric artery
SPA: Splenic artery
